# Heme catabolism and heme oxygenase-1-expressing myeloid cells in pathophysiology

**DOI:** 10.3389/fimmu.2024.1433113

**Published:** 2024-10-24

**Authors:** Francesca Maria Consonni, Martina Incerti, Milena Bertolotti, Giulia Ballerini, Valentina Garlatti, Antonio Sica

**Affiliations:** ^1^ Department of Pharmaceutical Sciences, University of Piemonte Orientale “A. Avogadro”, Novara, Italy; ^2^ IRCCS Humanitas Research Hospital, Rozzano, Milan, Italy; ^3^ Navita S.r.l., University of Eastern Piedmont A. Avogadro, Novara, Italy

**Keywords:** HO-1, immunosuppression, innate immunity, myeloid cells, immunemetabolism

## Abstract

Although the pathological significance of myeloid cell heterogeneity is still poorly understood, new evidence indicates that distinct macrophage subsets are characterized by specific metabolic programs that influence disease onset and progression. Within this scenario, distinct subsets of macrophages, endowed with high rates of heme catabolism by the stress-responsive enzyme heme oxygenase-1 (HO-1), play critical roles in physiologic and pathological conditions. Of relevance, the substrates of HO-1 activity are the heme groups that derive from cellular catabolism and are converted into carbon monoxide (CO), biliverdin and Fe2+, which together elicit anti-apoptotic, anti-inflammatory activities and control oxidative damage. While high levels of expression of HO-1 enzyme by specialized macrophage populations (erythrophagocytes) guarantee the physiological disposal of senescent red blood cells (i.e. erythrocateresis), the action of HO-1 takes on pathological significance in various diseases, and abnormal CO metabolism has been observed in cancer, hematological diseases, hypertension, heart failure, inflammation, sepsis, neurodegeneration. Modulation of heme catabolism and CO production is therefore a feasible therapeutic opportunity in various diseases. In this review we discuss the role of HO-1 in different pathological contexts (i.e. cancer, infections, cardiovascular, immune-mediated and neurodegenerative diseases) and highlight new therapeutic perspectives on the modulation of the enzymatic activity of HO-1.

## Introduction

The growing interest on the relevance of myeloid cell specialization in disease (1), is fueling new efforts to therapeutically re-educate their functions (2). Notably, macrophage phenotypic heterogeneity originates during fetal development from embryonic progenitors, which differentiate into self-regenerating subsets of tissue-resident macrophages (TRMs) and become essential for tissue homeostasis and repair (3). A list of abbreviations and acronyms used in the manuscript has been included in **Table 1**. In adults, blood monocytes are derived primarily from the bone marrow and orchestrate effector and repair functions of defense, also acquiring phenotypic traits of TRMs. In pathology, immunological stresses promote alterations of the myelopoietic output, defined as emergency (4), which lead to the generation of different myeloid populations endowed with specialized functions and distinct metabolic traits (5). This emergency response largely depends on inflammatory signals, which instruct differentiation and maturation of hematopoietic precursors, in a demand-adapted fashion (6). Although such heterogeneity may offer both beneficial and detrimental contributions to therapy, we are not yet able to select the myeloid phenotype with the greatest benefit. New studies are now delineating distinct myeloid subtypes, whose functional specializations appear to take on distinct pathophysiological meanings in the different pathologies, including cancer (7).

**Table 1:** List of abbreviation and acronymous used in the manuscript.

Abbreviation	Definition	Abbreviation	Definition
AMPK	5′-AMP-activated protein kinase	LL2	Lewis lung carcinoma
AD	Alzheimer's disease	LXR	liver X receptor
ALS	amyotrophic lateral sclerosis	M(Hb)	hemoglobin-stimulated macrophages
AngII	angiotensin II	MAPK	mitogen-activated protein kinase
AP-1	activator protein-1	MCP-1	chemoattractant protein 1
APOE	apolipoprotein E	Mhem	macrophages heme
Arg1	arginase 1	MI	myocardial infarction
ATF1	transcription factor 1	MN/MCA1	fibrosarcoma
B16/F10	melanoma	MS	Multiple Sclerosis
BMDMs	Bone marrow–derived macrophages	Mtb	Mycobacterium tuberculosis
BR	bilirubin	NF-κB	nuclear factor kappa-light-chain-enhancer of activated B cells
BV	biliverdin	NK	natural killer
CD163	hemoglobin/haptoglobin scavenger receptor	NO	nitric oxide
CNS	central nervous system	Nr4a1	nuclear receptor subfamily 4 group A member 1
CO	carbon monoxide	Nrf2	NF-E2-related factor 2
COPP	cobalt protoporphyrin IX	OVA	ovalbumin
CORMs	carbon-monoxide releasing molecules	PBMCs	peripheral mononuclear cells
CSF-1	colony stimulating factor 1	PD	Parkinson's disease
DAMP	Damage-associated molecular patterns	PD-1	programmed cell death protein 1
DCs	dendritic cells	PD-L1	programmed death-ligand 1
EAE	experimental autoimmune encephalomyelitis	PDAC	pancreatic ductal adenocarcinoma
EMT	epithelial-mesenchymal transition	PI3K	phosphatidylinositol 3-kinase
FPN	ferroportin	RA	rheumatoid arthritis
GBM	glioblastoma	RNS	reactive nitrogen species
GST	glutathione S-transferase	ROS	reactive oxygen species
Hb-Hp	hemoglobin haptoglobin	SARS-CoV-2	severe acute respiratory syndrome coronavirus 2
HD	Huntington's disease	SCD	sickle cell disease
HIF-1α	hypoxia inducible factor	SLE	systemic lupus erythematosus
HIV	Human Immunodeficiency Virus	SnMPIX	tin mesoporphyrin IX
*Hmox-1*	heme oxygenase 1 (gene)	SnPPIX	tin protophorphyrin IX
*Hmox-2*	heme oxygenase 2 (gene)	TAMs	tumor associated macrophages
HO-1	heme oxygenase 1 (protein)	TME	tumor microenvironment
HO-2	heme oxygenase 2 (protein)	TNF	tumor necrosis factor
IFN	interferon	TRMs	tissue-resident macrophages
IL	interleukin	VEGF	vascular endothelial ggrowth factor
IRF	IFN regulatory factor	ZnPPIX	zinc protoporphyrin IX
LDL	low-density lipoprotein		

The HO-1 enzyme encoded by the Hmox1 gene was first described in 1968 as a key microsomal component, which together with the HO-2 enzyme isoform, encoded by the Hmox2 gene, forms the heme oxygenase (HO) system involved in catalyzing heme oxidation and its related degradation. While Hmox1 can be induced throughout all tissue types but is most highly expressed within the spleen and liver, Hmox2 is constitutively and ubiquitously expressed, with specific enrichment in the testes and brain (8). Further, in contrast to HO-1, which is mainly involved in iron homeostasis, angiogenesis, mitochondrial function, and regulation of innate and adaptive immunity, regulating tissue responses to damage in pathophysiological states (9), HO-2 appears more as a physiological regulator of cellular functions and is involved in oxygen and redox sensing, neovascularization and neuroprotection (10).

**Figure 1:**
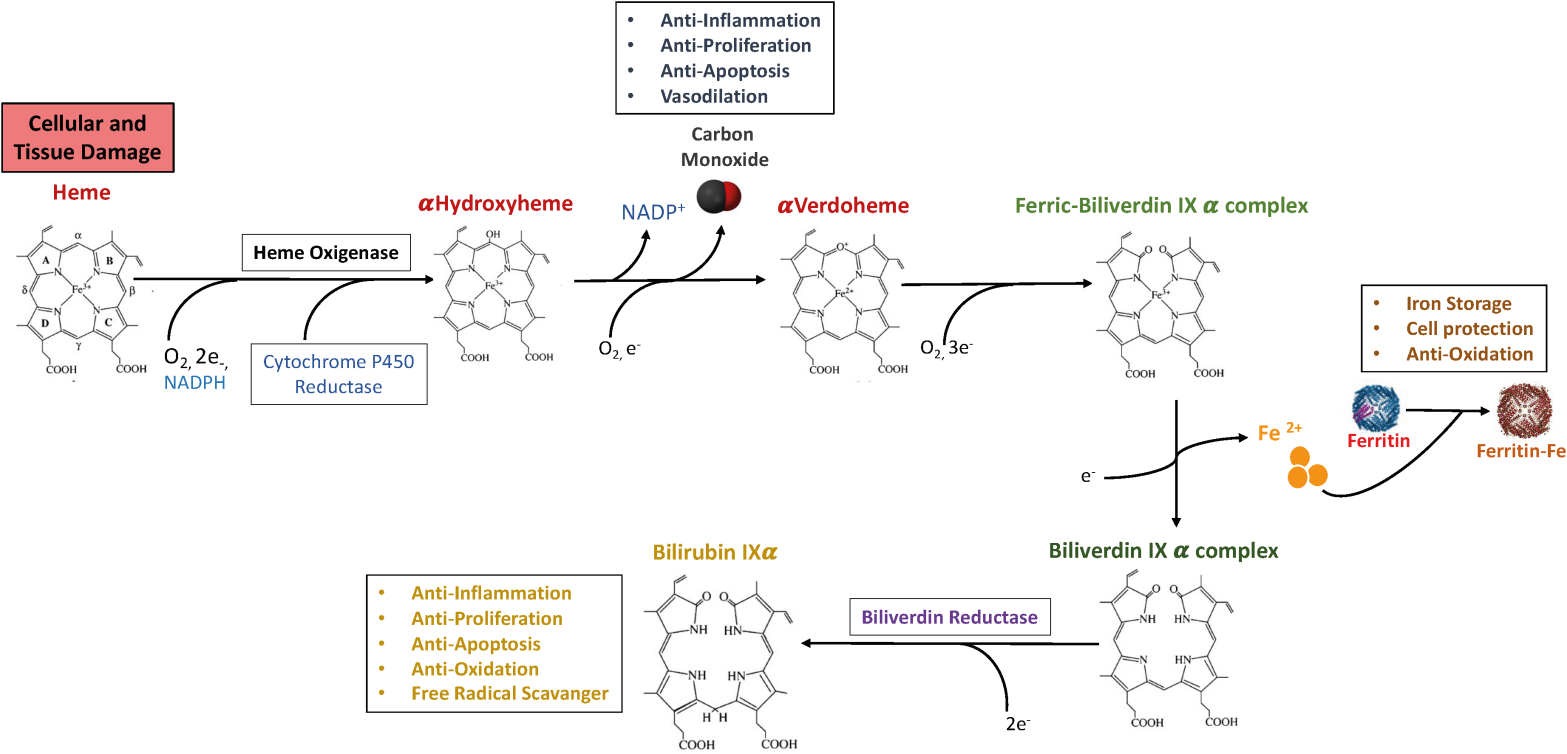
The heme oxygenase enzyme reaction and products. Heme is enzymatically degraded into biliverdin (BV), carbon monoxide (CO) and Iron. Biliverdin (BV) is subsequently transformed into bilirubin (BR) by biliverdin reductase (BVR). Iron can be bound by Ferritin, a protein able to store iron. The heme molecule provides a variety of fundamental biological functions as forming of various apo-heme proteins like hemoglobin, nitric oxide synthase and cytochromes. The HO-1-derived product CO could exert anti-inflammatory, anti-proliferation, anti-apoptosis, and vasodilation effects in immune system. BR can perform a crucial role in anti-inflammation, anti-proliferation, anti-apoptosis, anti-oxidation, and free radical scavenger. Moreover, iron-induced ferritin could play a cytoprotective, anti-oxidative and iron storage effect. A list of abbreviations and acronyms used in the manuscript has been included in **Table 1**.

The process of heme synthesis has been extensively reviewed 11–13). While erythrocytes produce around 85% of total heme content in the organism necessary for complete hemoglobinization, the predominant portion of the remaining synthesis occurs in the liver, which is highly enriched in cytochromes p450 (14). The biological activities and metabolism of free heme have been studied extensively, due to its hydrophobic and highly reactive properties, which confer it the ability to induce the formation of hydroxyl radicals, thereby promoting lipid oxidation, protein damage, and cell death (15). While hemoglobin heme undergoes synthesis in erythrocytes and ultimate degradation in the reticuloendothelial system (12), the final products of heme catabolism catalyzes the breakdown of heme into biliverdin, carbon monoxide (CO), and free iron, which display powerful anti-inflammatory and antioxidant potential (**Figure 1**) (12, 16). As depicted in **Figure 1**, cytochrome p450 reductase reduces the HO-1/ferrous heme complex, generating ferrous heme which binds and activates molecular oxygen through a protonation reaction, that in turn determines the formation of the reactive intermediate Fe3+-OOH and the formation of hydroxyheme. Next, oxygen activation promotes the conversion of ferric hydroxyheme to ferrous verdoheme and CO. A final round of oxygen binding and activation of Ferrous verdoheme determines the cleavage of the heme porphyrin ring and the generation of ferric iron biliverdin. Next, NADPH-cytochrome p450 reductase imposes the reduction of ferric biliverdin and the consequent release of free ferrous, as well as of biliverdin that is metabolized to bilirubin by biliverdin reductase (17). This cascade of reactions is thought to occur in respirating organisms whose cell types express and require the regulation of heme. Here, we review the homeostatic and immunoregulatory functions of HO-1, whose induction differentially affect the course of various pathologies.

## Role of myeloid heme oxygenase 1 in tumor progression

A vast literature points out that the inducible enzyme HO-1 plays a pivotal role in cellular adaptation to various stress factors, from oxidative insults to hypoxia, thus maintaining redox homeostasis and preventing cell damage through its cytoprotective, antioxidant and pro-surviving properties (18–20). As such, it is not surprising that HO-1 is widely expressed in cancer (i.e. glioblastoma, melanoma, bladder, breast, colorectal, renal cancer, prostate) (21) and that its overexpression correlates with cancer invasiveness, resistance to therapies and poor prognosis in different tumors (21–26). In particular, TCGA pan-cancer analysis shows that *Hmox-1* expression is significantly associated with epithelial-mesenchymal transition (EMT) in most tumors and accordingly, HO-1 target genes modulate adhesion, signaling, transport and other key cellular functions in neoplastic cells, promoting tumor cells proliferation and dissemination (27, 28). Indeed, heme and iron metabolism are key determinants of energy metabolism, hence affecting cancer cell functions and immune responses (29), as well as metabolic disorders (30).

In support of this conclusion, genetic polymorphisms of HO-1 promoter have been associated with an increased risk of cancer progression and a high degree of therapy failure (31). Moreover, alteration of the Kelch-like ECH-associated protein 1/nuclear factor erythroid 2–related factor 2 (Keap1/Nrf2) pathway, a major regulator of cytoprotective responses to oxidative that continuously targets Nrf2 for proteasome degradation (32), is also associated with tumor progression (33). Under stress conditions, the conformational change of Keap1 promotes the transcriptional activity of Nrf2 and consequently the expression of antioxidant genes such as heme oxygenase 1 (HO-1) (34). Recently, KEAP1 mutations were connected to adverse outcomes in NSCLC patients undergoing immunotherapy (33). Of relevance, Nrf2 is involved in the regulation of various molecules and enzymatic activities that control drug metabolism (35) as well as in multidrug resistance (36). In agreement, Di Biase et al. reported in the 4T1 breast cancer and B16 melanoma models that fasting mimicking-diet (FMD) caused reduction in HO-1 expression in cancer cells, sensitizing them to doxorubicin and cyclophosphamide, promoting intratumoral accumulation of cytotoxic CD8^+^ T cells and reducing tumor-associated Tregs activation (37).

Despite this evidence mainly poses for the protumoral role of HO-1, controversial anti-tumoral roles of HO-1 has also been reported (i.e. hepatocellular carcinoma, lung mucoepidermoid carcinoma) (38, 39), highlighting the complexity of the HO-1 activity in cancer and suggesting that HO-1 mediated functions are cell type, metabolism and TME dependent (23). These discrepancies also emerge from the specialized role that HO-1 displays during different stages of tumor development. In fact, while HO-1 deficiency in normal cells enhances DNA damage and carcinogenesis, HO-1 overexpression in cancer cells promotes cancer cell proliferation, survival and tumor angiogenesis (40).

### HO-1-expressing protumor myeloid cells

The tumor-promoting action of HO-1 is implemented by the biologically active catabolites of heme degradation not only through their action on tumor cells, but also hampering immune cells functionality and favoring pro-metastatic events (41). Borrowing from the physiological role of HO-1-expressing macrophages during the process of erythrophagocytosis (42), HO-1 expression has been frequently reported in malignant cells and tumor associated macrophages (TAMs) (7, 43–46). However, HO-1 can also be expressed by dendritic cells, regulatory T cells and endothelial cells (25, 47, 48). Thus, the tissue-specific contribution of HO-1 in tumor progression and as a therapeutic target still represents a hot undefined topic in cancer research. Within this scattered scenario, the major tumoral source of myeloid-related HO-1 are TAMs (7). These highly plastic cells are able to express distinct transcriptional and functional programs in response to the different cues in the TME, adopting distinct spectra of pro- or anti-inflammatory phenotypes (49, 50). Due to the breadth of its tumor-promoting roles and anti-inflammatory activities, HO-1 expression has been associated with biased M2-like TAMs and several recent reports proved its selective up-regulation in specific TAM subsets (7, 22, 44, 45, 51, 52). Reflecting the opposite and dynamic relationship intervening between therapeutic and pathological inflammation towards tumor development (53), the activity of HO-1 appears to be stage-dependent, with preventive function in the early oncogenic phases and of promotion action during tumor progression (54).

In preclinical models of Lewis lung carcinoma (LL2) and pancreatic ductal adenocarcinoma (PDAC), a distinct subset of HO-1^+^ F4/80^hi^ TAMs with an M2 phenotype co-expressing the fibroblast activation protein alpha (FAP^+^ HO-1^+^ TAMs) and representing 10% of the total TAMs population, has been described to be the major tumor source of HO-1 (51). Conditional ablation of FAP^+^ HO-1^+^ TAMs in an immunogenic ovalbumin (OVA)-expressing LL2 tumor, using diphtheria toxin in a bone marrow chimera of a FAP/diphtheria toxin receptor (DTR) transgenic mouse, or pharmacological inhibition of HO-1 with tin mesoporphyrin (SnMP), decreased LL2 and PDAC tumor growth, confirming the pro-tumoral and immunosuppressive role of TAMs-derived HO-1 (51, 55).

FAP^+^ TAMs have been described also in human (56) and murine breast tumors (44). By using the preclinical 4T1 model of breast adenocarcinoma, Muliaditan et al. (44) showed that an IL-6-dependent protumorigenic FAP^+^ HO-1^+^ TAM population expresses a cytokine profile similar to that which characterizes wound repair. Since inhibition of HO-1 results in a delay in wound closure (57), the action of HO-1 in tumorigenic processes is increasingly consistent with the Dvorak’s definition of tumors as “non-healing wounds” (58). Of note, FAP^+^ HO-1^+^ TAMs were predominantly located in the perivascular region of tumors and supported trans-endothelial tumor cells migration and metastatic spread. The study also demonstrated that the heme catabolite carbon monoxide (CO) directly facilitates tumor cells migration and, accordingly, SnMPIX-mediated pharmacological inhibition of HO-1 prevented metastatic spread (44).

In line, a distinct subset of bone marrow-derived M2 polarized F4/80^hi^ HO-1^+^ TAMs was recently described to play a specialized role in forming a pro-metastatic TME, promoting immunosuppression, EMT transition of angiogenesis and inhibition of T cell-mediated antitumor immunity (7). This population accumulates in the blood of cancer bearers (i.e. fibrosarcoma (MN/MCA1) and melanoma (B16/F10)) and preferentially localizes at the invasive tumor margins under the influence of NF-κB1/CSF1-R/C3a signaling axis, which converges on Nrf2 activation to support HO-1 expression. Importantly, pharmacological inhibition with zinc protoporphyrin IX (ZnPPIX) or myeloid-specific ablation of HO-1 blocked metastasis formation and improved the efficacy of anti-PD-1-mediated immunotherapy (7). Noteworthy, HO-1 expression in peripheral monocyte subsets as well as in tumor lesions discriminates survival among metastatic melanoma patients, suggesting HO-1 myeloid cells as a new prognostic indicator and a novel antimetastatic target (7).

In an aggressive spontaneous mouse model of breast cancer (*MMTV-PyMT*), TAMs were reported to be the major intratumoral source of HO-1, playing a crucial role in orchestrating immunosuppressive circuits that occur in the TME (22). Consistently, in this experimental setting, specific conditional ablation of HO-1 in the myeloid lineage improved the proportion of cytotoxic CD8^+^ T cells expressing IFNγ, granzyme B and TNFα, while pharmacological inhibition of HO-1, using SnMPIX, increased the antitumor activity of 5-fluorouracil (5-FU) in a CD8^+^ T cell-dependent manner (22). Importantly, HO-1 inhibition appeared as a potentially crucial immunotherapy target, hierarchically more important than PD-1. Moreover, treatment of *MMTV-PyMT* tumors with SnPPIX displayed increase response to chemotherapy compared to anti-PD-1 neutralizing antibody, suggesting that SnPPIX could be used as immune checkpoint therapy (22). The link between HO-1-expressing TAMs and cancer progression was further supported in immunogenic OVA-expressing murine thymic lymphoma model (EG/-OVA) (45). In this tumor, HO-1 is upregulated during the differentiation of monocytic Ly6C^hi^ cells into TAMs, becoming a key molecular effector of their immunosuppressive functions. Specific myeloid ablation of HO-1 or its pharmacological inhibition increased the anti-tumor response, improving the anti-tumor efficacy of a therapeutic anti-tumor OVA vaccine (45). It was also recently described an essential role of CX3CR1+ gut macrophages in resolving inflammation in the intestine, where they protect against colitis-associated cancer by regulating HMOX-1 expression (59).

Noteworthy, HO-1 could directly skew the polarization of macrophages (60); indeed, myeloid-specific ablation of HO-1 in bone marrow-derived macrophages (BM-DMs) treated with either M1- (LPS) or M2- (IL-4) polarizing signals produced an increase in the expression of M1 markers (CXCL10, IL-1b, MCP1), along with the decrease of M2 markers (Arg1 and CD163) (61). Other evidence shows in a murine breast cancer model (4T1) that following phagocytosis of cellular debris from tumor cells treated with chemotherapy (paclitaxel) TAMs upregulate the expression of HO-1, which in turn hinders M1 polarization and attenuates the response to chemotherapy. In contrast, genetic or pharmacological inhibition of HO-1 in TAMs reinstates M1-like polarization by restoring an immunogenic TME during chemotherapy, favoring the recruitment and activation of cytotoxic CD8+ T cells (62, 63). Along this line, Magri et al. reported that pharmacological inhibition of HO-1 in BM-DMs isolated from glioblastoma samples, with the HO-1 inhibitors ZnPPIX and OB-24, significantly reduced cell-to-cell- (i.e. PD-L1/PD-1) and soluble-dependent (i.e. IL-10) immunosuppressive mechanisms. Strikingly, HO-1 inhibition also prevented expression of immunosuppressive enzymes involved in amino acid catabolism (i.e. IDO1 and ARG2) (64). HO-1 inhibition by ZnPPIX was reported to repolarize M2-like protumor TAMs to antitumor M1-like macrophages also in the 4T1 breast cancer model (65). The influence of HO-1 on the TME immunoprofile was highlighted by Alaluf et al., who reported that myeloid-specific ablation of HO-1 induced global transcriptional and epigenetic alterations not limited to the conventional M1/M2 polarization state of TAMs, but rather leading to extensive dysregulation of the central molecular signature of the TME (45).

### The HO-1/CO pathway in cancer development

CO is a highly toxic gas due to its high affinity for hemoglobin, 250 times greater than oxygen. Due to its strong bond with heme iron, CO inhibits the transport of oxygen in the blood, while interacting with cytochrome c oxygenase and cytochrome p450 it inhibits cellular respiration and promotes tissue death (66). Despite the role of CO gasotransmitter has been the subject of various studies, its role in cancer is still largely unclear and both tumor-promoting and anti-tumor activities have been reported (67). It was demonstrated that CO directly modulates macrophage polarization *in vitro*, skewing their phenotype toward the anti-inflammatory one (68) and dampening their activation through M1-oriented signaling (i.e. TLR and MAPK) (69), with downregulation of the pro-inflammatory cytokines TNFα and IL-1βand increased IL-10 production (20). Consistently, in non-cancer settings, *in vitro* treatment of human monocyte-derived DCs with CO has been reported to block TLR3- and 4-induced phenotypic maturation and alloreactive T cell proliferation (70).

Accordingly, *in vitro* treatment of macrophages with carbon-monoxide releasing molecules (CORMs) enhances STAT3/STAT6 activation to induce their anti-inflammatory phenotype (7). In contrast, by using the *in vivo* A549 lung carcinoma model, Nemeth et al. showed that exposure to low doses of exogenous CO polarizes macrophages toward a pro-inflammatory M1-like phenotype through ROS-dependent activation of MAPK/Erk1/2-c-myc pathway, which negatively regulates HO-1 expression leading to an anti-tumor effect (71).

HO-1 catabolites can also influence the activation states and phenotype of a variety of cell populations in the TME, enhancing immune evasion and ultimately contributing to tumor progression (41). In this regard, CO inhibits the maturation of dendritic cells (DCs) by maintaining them in a pro-tolerogenic state and supporting HO-1 expression through increased IL-10 production (47, 70). Consistently, HO-1 expression contributes to imprint a pro-tolerogenic signature of DCs (72). The product of heme degradation biliverdin has also been reported to induce IL-10 production in macrophages through a PI3K-Akt dependent pathway (73), while both CO and bilirubin down-regulate expression of MHCII on DCs, restraining their ability to present antigens CD4^+^ T cells (70, 74).

The HO-1/CO axis has also been described to play a critical role in FoxP3-mediated immune suppression (48, 75). In glioma patients, HO-1 mRNA expression has been linked to Foxp3 induction in infiltrating CD4^+^CD25^+^ Treg cells and correlated with glioma progression and grading (48), while in the preclinical model of malignant glioma the expression of HO-1 improved the survival of Tregs in the hypoxic regions of the TME (75). HO-1-mediated suppression of T cell proliferation is not only associated with Treg cell expansion, as growing evidence demonstrates the ability of HO-1/CO to directly block the expansion of effector T cell populations (76). CO has been described to suppress the secretion of IL-2, a cytokine required for T-cell entry into the cell cycle (76) and CO-mediated inhibition of CD3-activated T cell proliferation is highly dependent on caspase-3 and -8, which are regulated by *p21^Cip1^* (77). HO-1 was also reported to suppress natural killer (NK) activation and their effector functions, through interfering with the expression of activator receptors (NKG2D, NKp46 and NKp30), as well as blocking their ability to secrete IFNγ and TNFα (78). In acute myeloid leukemia (AML), HO-1 has also been described to prevent cytotoxic effects of NK by inhibiting CD48 expression, a ligand of the NK-activating receptor 2B4, through Sirt1-H3K27-dependent pathway (79). Numerous studies also highlight an intriguing direct pro-metastatic role of HO-1/CO on tumor cells (28). CO has been reported to increase glioma cell survival and migration (80–82), while HO-1 silencing using small interfering RNAs (siRNAs) causes downregulation of VEGF-induced vimentin and endothelial cell proliferation, resulting in impaired angiogenesis and reduced tumor progression (81). In agreement, myeloid expression of HO-1 facilitates tumor metastasis by promoting the formation of a premetastatic niche and increasing tumor colonization at the metastatic site (82).

The preclinical and clinical observations available today are consistent with the crucial role played by HO-1 in arresting cancer immune recognition and supporting tumor progression and pharmacological targeting of HO-1 and its catabolites is acquiring solid confirmation as promising anticancer therapy.

## HO-1 in immune-mediated diseases

HO-1 plays a critical role in the maintenance of immune-homeostasis, as this enzyme elicits a strong impact in both innate and adaptive immune responses. In particular, the expression of heme oxygenase by immune cells and, later on, its products of reaction exert extensive antioxidant and anti-inflammatory properties associated with beneficial outcomes in inflammatory disorders (18). Consistently, a chronic inflammatory condition is observed in *Hmox1*-knockout mice, paralleled to an accumulation of polymorphonuclear cells, increased number of monocytes in the spleen and lymphocyte count in peripheral blood (83).

In accordance with the beneficial role of this cytoprotective enzyme, different polymorphisms in the *Hmox1* promoter region determine different levels of HO-1 induction, associated either with protective functions or with increased susceptibility towards autoimmune and inflammatory diseases, such as rheumatoid arthritis (RA) or systemic lupus erythematosus (SLE) (84). Consolidated evidence also supports a key role of HO-1 in controlling intestinal and lung inflammation (85, 86). Supporting this evidence, the presence of a long allele in a (GT)_n_ microsatellite polymorphism in *Hmox-1* gene promoter leading to decreased expression of HO-1, gives rise to a pronounced risk and higher susceptibility to develop autoimmune disease, such systemic lupus erythematosus (87). On the other hand, the presence of a short (S) allele, provoke an increase in the production of HO-1 leading to a protection in developing immune-mediated disorders (18, 88). The up-regulation or the downregulation of this enzyme in immune-mediated disorders therefore makes it a possible inflammatory marker for autoimmune diseases (83).

In RA patients a significant increase of HO-1 levels was observed in synovial fluid and peripheral monocytes, as an adaptive mechanism for limiting inflammation and toxicity (89). Furthermore, induction of HO-1 in SLE confers an anti-inflammatory phenotype to monocytes and DCs, while myeloid cells in these patients show downregulated HO-1 levels, suggesting that its deregulation is involved in disease progression (90). According to the HO-1 protective functions, the anti-inflammatory effects of IL-10 in macrophages appear to be mediated via induction of HO-1 (91, 92).

HO-1-derived CO plays a crucial immunomodulatory role, by acting on different immune cells, including dendritic cells, macrophages and regulatory T cells (93). Indeed, HO-1 induction fosters the polarization of macrophages towards an anti-inflammatory M2-like profile and promotes a tolerogenic phenotype in DCs (83, 94), both reducing the activation of T cells and favoring the Treg differentiation, resulting in the suppression of autoreactive responses (47). All this reveals the strong impact that HO-1 elicits in the context of immune-mediated diseases, as well as its centrality for the maintenance of immune homeostasis.

### HO-1 in inflammatory bowel diseases

Accumulating evidences indicate that induction of HO-1 expression act as an endogenous defensive mechanism to reduce tissue injury in the intestinal tract associated with chronic inflammatory conditions (i.e. Inflammatory bowel disease/IBD), including Crohn’s disease (CD) and Ulcerative Colitis (UC) (95). Chronic intestinal inflammation is promoted by different conditions, including infections and autoimmune diseases, and is characterized by massive immune cell infiltration, edema and alterations of the epithelium structure (96). Of note, HO-1 is shown to be transcriptionally induced in the intestinal tract in response to oxidative stress (97, 98) and pharmacological evidence supports the protective role of HMOX1 in intestinal inflammation. In the model of colitis induction by administration of Dextran Sulfate Sodium (DSS), administration of HMOX1 inductor/activator CoPP significantly reduced the intestinal histological damage as compared to control animals (99). This protective response was mimicked by administration of the HMOX1 inducer hemin, which also reduced number of Th17 cells and increased number of Treg cells in mesenteric lymph nodes (MLN) and spleen (100).

Of particular note, a strong association between the development of IBD and the immune response to microbial infections has been described, the mechanistic basis of which are still unclear. In this context, it is important to note that HMOX1-like enzymes are expressed in bacteria and CO can therefore directly interact with the heme groups of the bacterial electron transport chain (101). These evidences support an increasingly central role of HO-1 and CO in the cross-talk between the microbiota and the mucosal immune compartment, suggesting HO-1 as a new therapeutic target for inflammatory bowel disease (102).

### HO-1 in inflammatory lung diseases

Mounting evidence indicates that in various pulmonary diseases, such as acute respiratory distress syndrome (ARDS) and interstitial lung disease (ILD), an increase in HO-1 expression in alveolar macrophages reflects the activation of an M2 macrophage phenotype polarized against oxidative stress (103, 104). In agreement, serum heme oxygenase (HO)-1 level has been indicated as a potential marker of acute progression of interstitial lung disease (ILD) (105). Preclinical models of acute lung injury (ALI) (i.e., hyperoxia, sepsis, and ventilator-induced lung injury) have highlighted the protective effects of the HO-1/CO system (93). In the lipopolysaccharide (LPS)-induced lung injury mouse model, up-regulation of HO-1 by gene transfer limited neutrophil influx and pro-inflammatory response, protecting against ALI. This protective effect was reproduced by treatment with CO (250 p.p.m.), resulting in downregulation of the p38 MAPK-dependent pro-inflammatory cytokine response and upregulation of IL-10 (20). Furthermore, gene transfer of HMOX1 also protected against ALI induced by influenza A virus infection, reducing the influx of inflammatory cells (106). Neutrophil accumulation is a major cause of damage in many lung diseases, including cystic fibrosis (CF). Macrophages from patients with CF have been observed to exhibit a defect in the heme oxygenase-1 (HO-1)/carbon monoxide (CO) pathway. Recent evidence has shown that systemic administration of PP-007, a CO releasing/O2 transfer agent, rescues the PI3K/HO-1 axis in CF macrophages and decreases the hyperinflammatory response to LPS, suggesting the HO-1 pathway as a potential candidate therapeutic target (107). This is consistent with the abundant expression of HO-1 observed in monocytes/macrophages responsible for resolving the inflammatory response, whose dysregulation leads to hyperinflammation in CF, asthma, COPD and fibrotic lung diseases (108, 109). Human respiratory syncytial virus (hRSV) is the leading cause of severe lower respiratory tract infections in children. Upregulation of HO-1 with cobalt metalloporphyrin protoporphyrin IX (CoPP) in hRSV-infected mice significantly reduced disease-related body weight loss and induced an upregulation of IFN-α/β in the lungs, indicating that HO-1 plays an important role in the development of the antiviral type I IFN response in the airways (106). Despite no direct evidence has been collected on the connection between HO-1 and COVID-19 severity, HO-1 expression has been reported in these patients and its serum levels have been proposed as a useful marker for evaluating disease severity (110, 111).

## HO-1 in neurodegenerative disorders

The upregulation of HO-1 in the central nervous system (CNS) constitutes a mechanism of cellular adaptation to stress, which crucially mediates the resolution of neuroinflammation through the activation of antioxidant, antiapoptotic and anti-inflammatory properties (112). In agreement, much evidence indicates a cytoprotective effect of HO-1 in the pathogenesis of neurodegenerative diseases, including amyotrophic lateral sclerosis (ALS), including Alzheimer’s disease (AD), Huntington’s disease (HD) and Parkinson’s disease (PD) (113). Accordingly, while Nrf2-dependent activation of HO-1 is related to a protective and beneficial effect in neurons, this pathway drives the transcription of genes involved in iron quenching, chelation and transport (114, 115), as well as in prevention of lipid peroxidation (116, 117), resulting cytoprotective action on cells and tissues.

In contrast, in conditions of excessive stress, the high production of this enzyme, as well as its metabolites by neuronal or immune cells, favors the deposition of mitochondrial iron and the depletion of bioenergy, which favors the development of degenerative diseases such as multiple sclerosis (MS) or Alzheimer’s disease (118).

Abnormal pathways of HO-1 induction via unconventional signaling pathways have been investigated. Among these, accumulation of p62, also known as sequestosome 1 (SQSTM-1) and A170 (119), sequesters Keap1 into aggregates, resulting in the inhibition of Keap1-mediated Nrf2 ubiquitination and its subsequent degradation by the proteasome (120). Consistently, in neurodegenerative diseases it was observed an imbalance in the signaling pathway of HO-1 induction, leading to the loss of normal tissues and immune homeostasis, cellular deterioration and progressive development of neurodegeneration (114). In “stressed” astrocytes, excessive HO-1 activity can lead to mitochondrial iron sequestration, macroautophagy, pathological iron deposition, and bioenergy depletion, which contribute to the development of neurodegenerative diseases such as Alzheimer’s disease (AD), Parkinson’s disease (PD), and Huntington’s disease (HD) (121).

### HO-1 in multiple sclerosis

Multiple Sclerosis (MS) is a central nervous system demyelinating neurodegenerative immune-mediated disease characterized by the production of inflammatory lesions in the brain, optic nerve and spinal cord (122). This disorder is also marked by a pro and anti-inflammatory cytokine imbalance, which leads on one side to a progressive degeneration of oligodendrocytes and axons, from another to the proliferation of astrocytes (114). The importance of an equilibrium in the expression of HO-1 appears as a pivotal requirement for the maintenance of protective effect and immune homeostasis preventing the progression of the disease. In the early stage of muscular atrophy, the enzyme plays the role of essential regulator of cytoprotective responses (123).

It was reported that the excessive downregulation of Nrf2/HO-1 signaling pathway substantially enhances neuroinflammation and immune dysregulation associated with oligodendrocyte loss, advancing the stage of disease (114). Moreover, a severe reduction of HO-1 expression in peripheral mononuclear cells (PBMCs) from MS patients was demonstrated during the exacerbation of this disorder (124). The Experimental autoimmune encephalomyelitis (EAE) model has been frequently used to better understand the dual and intricate role of HO-1 in inflammation and its contribution to the immune system in MS (123, 125). It was observed that during the acute phase of disease, EAE mice show increased levels of HO-1 expression in microglia, astrocytes and oligodendrocytes (126). Furthermore, the chronic and constant upregulation of HO-1 by oligodendrocytes has been associated with a damage in glial cells, leading to induction of cell death and severe inflammation. Moreover, the plaques deposed in the spinal cord, during the progression of the disease, show a strong mitochondrial iron deposition induced by the secretion of IL-1 and TNF and provoked by the elevated expression of HO-1 (118).

If on one side the chronic over-expression of HO-1 results detrimental, on the other *Hmox1-*deficient mice showed an aggravated disease, because of the absence of cytoprotective effects elicited by the HO-1\CO axis. Interestingly, the protective features of HO-1, and in particular of CO, in MS has been associated with the inhibition of both CD4^+^ and CD8^+^ T cell activation and MHC II expression by antigen-presenting cells, including DCs, microglia and infiltrating macrophages (126). Induction of HO-1 expression by iron-containing porphyrin hemin and treatment with the CO donor CORM-A1 protects EAE mice from acute disease, showing improvement in clinical score and reduced incidence of disease infiltration by polymorphonuclear cells (123). Although the mechanism of protection of HO-1 in EAE remains to be investigated, the immunomodulation and cytoprotective properties it exerts on the central nervous system likely provide a preventative role in neuroinflammatory diseases.

### HO-1 in Alzheimer’s disease

Alzheimer’s disease (AD) is a neurodegenerative disorder characterized by a set of brain lesions caused by the accumulation of different proteins such as the β-amyloid in fibrillary plaques, which give rise to a severe state of inflammation and consequent tissue damage (127). In this setting, the maintenance of the expression’s balance in HO-1 signaling pathway appears crucial in controlling the disease progression. Accordingly, in neuronal cells this enzyme plays a protective role, mediating the conversion of prooxidant heme into its degradation and antioxidant products biliverdin (BV) and bilirubin (BR), which are crucial in the promotion of an appropriate tissue redox microenvironment (112, 128). It is established that HO-1 is over-expressed in the brain of AD patients, mainly in the hippocampus cerebral cortex, and is co-localized to neurons and neurofibrillary tangles. During acute brain damage this cytoprotective enzyme can be subjected to a protracted up-regulation leading to a detrimental effect for the neuronal tissue (115). The accumulation of mitochondrial iron, enhanced by glial HO-1 activity, leads to toxicity, protein accumulation and neuronal death. Further, the abnormal brain iron mobilization may amplify the oxidative stress and contribute to the progression of AD (129). Supporting the importance of the homeostatic equilibrium in HO-1 expression in AD, recent studies demonstrated that inhibition of HO-1 up-regulation in microglia reduces inflammation in brain lesions (118). Finally, the detrimental upregulation of HO-1 in cerebral AD patients, compared to age-matched non-dementia individuals, makes this enzyme a possible biomarker and therapeutic target for AD (130).

### HO-1 in Parkinson’s disease

Parkinson disease (PD) is a neurodegenerative disorder characterized by the progressive loss of dopaminergic neurons in substantia nigra, linked to the chronic movement disorder. Also, neuroinflammation and the presence of Lewy bodies, formed by the accumulation of intracellular aggregation of α-synuclein protein, result dangerous for brain loss of function (131, 132). As for other neurodegenerative diseases, also in PD patients the role of HO-1 has been studied for a long time. Of relevance, HO-1 appears overexpressed both in brain and plasma of Parkinson patients and its upregulation by astrocytes promotes the α-synuclein production, with consequent brain impairment and toxicity (115, 118). Lewy bodies contain a huge amount of iron and the overexpression of HO-1, together with its metabolites, contributes to the increase in its concentration. Augmented free iron is also observed in microglia and substantia nigra of PD patients leading to an impairment of cognitive abilities and neuronal iron-induced cell death, with progression of the disease (133). Consistently, it is observed that the upregulation of Nrf2 triggers with cerebral injury and disease progression, making high the expression of HO-1 (134).

## HO-1 in cardiovascular diseases

In spite the decline in mortality observed in recent decades, cardiovascular diseases still remain the main cause of death worldwide (135) and represent a urgent unmeet clinical need. Physiological HO-1 expression levels are usually low in most body areas, but increase in response to pathological stimuli, to buffer ongoing inflammation. This key role of HO-1 in tissue homeostasis has been documented by various studies and, remarkably, its cardioprotective effects emerge through its ability to regulate inflammatory processes and mitochondrial functions (30), ultimately mitigating damage to the cardiovascular system (136). Macrophages represent a functionally plastic (137) homeostatic population present in virtually all tissue, where they maintain proper organ functions, in part by recycling iron, regulating heme acquisition and decomposition (138). Macrophages are claimed as central players in the pathogenesis of various cardiovascular diseases (i.e. atherosclerosis, thrombosis and myocardial infarction), through local production of inflammatory cytokines and factors leading to oxidative stress (139). However, their ability to express HO-1 ensures antioxidant defenses and provides cardioprotective and reparative activities (140). As such, pathways of macrophages differentiation and identification of HO-1-expressing subsets may provide new strategies to control tissue injury and/or repair.

### HO-1 shapes the anti-inflammatory phenotype of macrophages in CDVs

HO-1 expression by macrophages critically integrates their M2 polarized phenotype, with specialized anti-inflammatory functions (60). Previous studies have demonstrated that HO-1 null macrophages display increasing levels of ROS, enhanced release of proinflammatory mediators and increased levels of oxLDL scavenger receptor A (SR-A) expression (141). Moreover, in response to bacterial products (i.e. lipopolysaccharide) HO-1 is recruited to the cell membrane caveolae through p38 MAPK-dependent mechanism, blocking the activated proinflammatory signaling cascade (142). Moreover, stimulation of macrophages with the anti-inflammatory interleukin-10 (IL-10) activates HO-1 expression in a p38 MAPK-dependent manner (92), while the protective activity of IL-10 in response to LPS stimulation is significantly abrogated after the administration of an HO-1 inhibitor. This evidence corroborates the hypothesis that engagement of HO-1 in response to IL-10 is an integral part of the resolution phase of the inflammatory response (143).

The idea of targeting HO-1 in the myeloid cell compartment appears advantageous because the main functions of the heme-HO-1 system have been highlighted in the myeloid-mononuclear system (7, 144, 145) and because cardiac healing after myocardial ischemia depends on the recruitment and local expansion of myeloid cells, particularly macrophages (146). Macrophages play a key role in the physiological processes of the cardiovascular system, such as cardiac contraction (147) and control of blood pressure (148), as well as in pathology, including clearance of necrotic tissue, tissue remodeling and self-renewal (149). Consistently, hemin-induced HO-1 expression provides cardioprotective activity, attenuating ischemic-induced cardiomyocytes senescence and preventing myocardial infarction (150). In agreement, mice deficient for Bach1, a negative regulator of the HO-1-inducing transcription factor Nrf2 (151), have dramatically higher HO-1 expression in the heart and display resistance to ischemia-reperfusion-induced myocardial injury (61, 152). The role of HO-1 in alternative M2 activation of macrophages was further confirmed in Bone marrow–derived macrophages (BMDMs) from myeloid-specific *Hmox1*–knockout mice, that showed increased expression of proinflammatory M1-related mediators as CXCL10, IL-1β, and monocyte chemoattractant protein 1 (MCP-1), along with increased M2-related markers (i.e. Arg1 and CD163) (61). The scavenger receptor CD163, in particular, mediates the internalization and clearance of hemoglobin haptoglobin (Hb-Hp) complexes, thus providing a fundamental step of heme metabolism, and is mostly expressed by macrophages (153).

Of relevance, the chemokine ligand CXCL4/PF4 inhibits CD163 in macrophages, compromising their phagocytic function and exacerbating the progression of atherosclerosis (154). However, alternatively activated, hemoglobin-scavenging CD163^+^ macrophages are present within atherosclerotic lesions and were also proposed to promote angiogenesis, vessel permeability, and leucocyte infiltration, via the CD163/HIF1α/VEGF-A pathway, thereby exacerbating plaque progression (155). Of note, CD163 exists also as soluble form of CD163 (sCD163), whose levels are induced by Inflammatory factors, including LPS, through activation of tumor necrosis factor‐α–converting enzyme (TACE/ADAM) metallopeptidase (156). Importantly, sCD163 levels were recently associated with established CVD risk factors and with carotid intima media thickness (157).

### HO-1 in ischemic conditions

The heart is enriched in the number of mitochondria due to its high metabolic demand, making it particularly sensitive to oxidative stress (158). Dysfunctional coronary blood flow following a heart attack usually leads to a hypoxic condition that promotes greater release of free radicals and causes ischemic heart disease, that may activate a persistent inflammatory condition fueling oxidative damage and the development of heart failure (159). In response to oxidative stress, an activated transcription factors signatures (i.e. the nuclear factor erythroid 2-related factor 2/Nrf2, NF-κB, AP-1 and Bach1) leads to HO-1 upregulation (160), likely involved in the defense against pathophysiological stress (161). In particular, Nrf2 binds to the Maf proteins and activates the antioxidant response element (ARE), sustaining and triggering the expression of HO-1 (162). Nrf2 has been appointed as one of the key modulators of related genes involved in the antioxidative process, as glutathione S-transferase (GST), γ-glutamyl cysteine synthetase (γ GCS) and HO-1 (163). During ischemic heart injury, neutrophils are rapidly recruited to the injured myocardium to initiate the inflammatory phase. The transition to resolution is promoted by macrophage efferocytosis of dying neutrophils, characterized by a time and context-dependent phenotypic M2 polarized switch, toward repair and resolution; Treg release of anti-inflammatory signaling also facilitates resolution (164). Importantly, mice with cardiac-restricted HO-1 overexpression resisted ischemia/reperfusion (I/R) injury, with improved contractibility and reduction of infarct size, oxidative damage, inflammatory cell infiltration and apoptosis (150).

Metabolites resulting from heme degradation, bilirubin and biliverdin (165), are reducing species and powerful antioxidants, mostly produced in the spleen following hemoglobin degradation by HO-1 (113). Coherently, low levels of BR correlate to cardiovascular risk, such as ischemic heart disease and hypertension (166).

### HO-1 in myocardial infarction

Myocardial infarction is the most acute manifestation of ischemic heart disease and one of the most life-threatening cardiovascular emergences (167). Risk factors also include arterial hypertension, smoking, dyslipidemia, diabetes mellitus, inactivity and early development of atherosclerosis, while elevation of myocardial infraction is found to be one of the cardiac emergency encountered with COVID-19 pandemic (168). ROS are involved in pathological myocardial dysfunction and their elimination through the antioxidant activity of HO-1 can be considered a potential therapeutic approach. Various cell types contribute to the triggering of sterile inflammation during tissue injury and myeloid cells, particularly resident cardiac macrophages of the myocardium, are involved in the regulation of primary innate immune response, stimulated by the activation of PRRs by Damage-associated molecular patterns (DAMPs). This release activates transcriptional factors, such as IRF, NF-κB and AP-1, causing the production of proinflammatory molecular mediators in the damaged heart (169). Suppression of these transcriptional pathways changes the macrophage phenotype favoring an anti-inflammatory M2-like polarization state, expressing pro-angiogenic properties, reducing pathological cardiac remodeling and preserving the ventricular rupture.

During MI, the recruitment of neutrophils and macrophages, enabled by the up-regulation of specific chemotactic signals of the CXC (CXCL2 and CXCL5) and CC (CCL2 and CCL5) chemokine families, guarantees the first line of defense (170, 171). More in-depth studies have shown that the healing process occurring after MI involves the early recruitment of inflammatory Ly-6C^high^ and the subsequent accumulation of reparative Ly-6C^low^ monocytes/macrophages. Such transition was shown to be controlled by the Nuclear Receptor Subfamily 4 Group A Member 1 (Nr4a1), which crucially modulates both the early Ly-6C^high^ monocyte inflammatory and the later Ly-6C^low^ macrophage reparative phases in the infarcted myocardium (172). It has also been observed that the ischemic myocardium accumulates Ly6C^high^ monocytes in greater numbers than their availability in circulation and that their main source is the spleen (173). Of relevance, the post-ischemic influx of Ly6G^+^ neutrophils and Ly6C^high^ monocytes is further exacerbated in *Hmox1*-deficient hearts, while treatment with recombinant adeno-associated virus (rAAV)-encoding human *Hmox-1* reverses such phenotype and attenuates post-ischemic inflammation, in a murine ischemia/reperfusion model (174). Consistently, HO-1 knock-out mice showed increased tissue dysfunction post-MI, positively correlating with a higher number of circulating Ly6C^high^ monocytes and increased number of proinflammatory macrophages in the damaged cardiac tissue (175).

Strikingly, in neonatal mice, the heart can regenerate fully, without scarring following MI (176). Interestingly, embryonic-derived macrophage subsets (MHC-II^low^CCR2^−^ and MHC-II^high^CCR2^−^) resident in the adult heart and owning tissue repair properties are lost after adult cardiac injury and are replaced by inflammatory CCR2^+^ monocyte-derived macrophages that interfere with the angiogenic process and the restoration of cardiomyocytes, thus establishing a reduced regenerative potential (177). This evidence appears in line with the observation that proinflammatory monocytes and monocyte-derived macrophages support the reduction of tissue-resident macrophages (176). Of note, after 3 weeks of MI, *Hmox1*-deficient mice express a pool of blood monocytic cells associated with expansion of cardiac macrophages overexpressing the CD11c marker (175), previously shown to release large amounts of inflammatory cytokines (i.e. IL-1β, TNF-α and IFNγ).

### HO-1-expressing macrophages in atherosclerosis

Coronary heart diseases are clinical manifestations of atherosclerosis, a process characterized by the development of atherosclerotic plaques that can lead to obstruction coronary arteries, ischemia of the cardiac tissue and myocardial infarction. Such process is supported by circulation of elevated levels of low-density lipoprotein (LDL), undergoing oxidization and accumulating at the level of the intima wall of the vessels. Uptake of oxidized LDL by monocyte-derived macrophages and smooth muscle cells leads to the formation of foam cells which accumulate lipid droplets and crucially contribute to development of atherosclerosis. Infiltrating monocytes are chemoattracted in the inflamed intima, migrating through the vascular wall and generating the primary atherosclerotic lesion, also identified as fatty streak (178).

Activated inflammation at the plaque level releases a cohort of inflammatory mediators that can induce cell death and the development of a central necrotic core, which favors plaque rupture (178). Based on this, emerging therapeutic strategies aim to target and mitigate inflammatory circuits that support plaque development and fragility. Several reports proved the HO-1 expression in atherosclerotic plaques (141), as well as its role in the early stages of atherosclerosis. This is in line with the detrimental role exerted by oxidative stress on the arterial wall in proatherogenic conditions (i.e. diabetes, hypertension or hyperlipidemia) (179). In agreement, it was shown that increased HO-1 activity markedly reduces the chemotactic recruitment of monocytes after exposure to oxidized LDL. It has in fact been verified that the induction of HO-1 by pharmacological or gene transfer approaches may prevent atherogenesis in preclinical hypercholesterolemic murine model (180). In analogy, genetic ablation of HO-1 amplified the development of atherosclerotic lesions and promoted their progression, thus confirming the protective role of HO-1 against atherogenesis (181).

In this scenario, a number of reports suggest that release of heme from intraplaque hemorrhage induces an atheroprotective macrophage phenotype (182–185); indeed, in the complex atherosclerotic plaques’ microenvironment, macrophages are simultaneously exposed to a variety of stimuli and, accordingly to their known functional plasticity, they polarize into multifunctional subsets which collectively cooperate to determine the dynamic evolution of the inflamed plaque, thus influencing atherosclerosis progression (184, 186, 187). In particular, in addition to the continuum of macrophage polarization states described within the M1 and M2 functional extremes (137), plaque-specific macrophage phenotypes, including Mhem and M(Hb) macrophages, related to the presence of hemoglobin and erythrocytes, have been recently identified, which play atheroprotective roles by preventing foam cell formation and lipid accumulation, as they highly express genes associated with reverse cholesterol transport, such as liver X receptor α and β (LXR-α; LXR-β) and the ATP-binding cassette transporters ABCA1 and ABCG1 (188–190). Due to the increased expression of molecules involved in cholesterol efflux, M(Hb) macrophages are characterized by low levels of lipid accumulation. In particular, M(Hb) macrophages typically express high levels of mannose receptor CD206 and the scavenger receptor for the hemoglobin/haptoglobin (Hb/Hp) complex CD163, participating in the hemoglobin clearance after plaque hemorrhage. These macrophages are described to produce both anti- (IL-10, IL-1Ra) and pro-inflammatory (VEGF, IL-1β) cytokines, while producing less ROS than other macrophage subtypes and display low iron accumulation because of the upregulation of ferroportin (FPN) (191).

After endocytosis of the Hb/Hp complex and erythrocytes, the released heme group primes intraplaque macrophages polarization toward a Mhem phenotype, with consequent activation of the 5′-AMP-activated protein kinase (AMPK) and the downstream transcription factor 1 (ATF1) (188, 189). Activation of the ATF1/MAPK signaling pathway therefore leads to the expression of HO-1, LXR-α/LXR-β, and ABCA1/apolipoprotein E (APOE) cascade, preventing foam cell formation. Thus, since iron levels in macrophages may drive cholesterol efflux, targeting macrophages environment manipulating iron levels and/or iron metabolism-related molecules could interfere with the generation of foam cells and the development of atherosclerosis.

Conversely, proatherogenic roles of CD163^+^ macrophages are also reported (155, 192, 193); indeed, in human and mouse atherosclerotic lesions M(Hb) CD163^+^ macrophages are also associated with promotion of angiogenesis, vessel permeability, and leucocyte infiltration through a mechanism involving Hb/Hp/CD163/HIF1α-mediated VEGF induction (194). Therefore, the plasticity of these cells makes the scenario more intricate, suggesting that the complete definition of the role of macrophage subpopulations in plaque needs to be further clarified.

### HO-1-expressing macrophages in hypertension

Arterial hypertension is associated with an increased risk of cardiovascular disease (CVD) and is responsible for more than 10 million deaths every year (195, 196). While endocrine and renovascular disorders are responsible for secondary hypertension in 5 and 15% of hypertensive patients, primary hypertension refers to patients in which no underlying cause has been found (197). Despite its clinical relevance, the causes for its occurrence still remain unclear, although the role of angiotensin II (AngII) in its development has been described. In fact, AngII, via its AT_1_ receptor, promotes cell growth, inflammation, vasoconstriction, apoptosis, production of extracellular matrix components and the release of ROS (9, 198). Specifically, the increase in the renin-angiotensin system and sympathetic activity contributes to the macrophage mobilization and to its polarization towards the pro-inflammatory phenotype (199). A low degree of inflammation facilitates vascular oxidative stress, leading to the vascular alterations accounting for increased peripheral vascular resistance (200, 201). The regulatory role of heme availability for the synthesis of enzymes such as cyclooxygenase or nitric oxide synthase, both involved in hypertension development, seems to be responsible for many of the beneficial effects of HO-1. Oxidative activity, in fact, activates cyclooxygenases (COX), with the consequent production of prostaglandins and thromboxanes, both contributing to vascular alterations and enhances inflammatory responses (202). HO-1 has been shown to protect against oxidative and inflammatory insults in hypertension, reducing organ damage and blood pressure, not only by its expression at the vascular level, but also by shifting macrophages toward the anti-inflammatory phenotype (203, 204). Moreover, the antioxidant, anti-inflammatory, antiapoptotic, and antiproliferative effects of the end products of HO-1 activity would also provide protective functions (199). In particular, bilirubin (BR) is one of the most powerful plasma scavenger of ROS and RNS, inhibiting lipid peroxidation and peroxynitrite-mediated oxidations, protecting against H2O2 toxicity and increasing NO half-life (205–207). Coherently, an inverse relationship between plasma BR levels and systolic blood pressure was reported (208, 209). Consequently, it was suggested that BR might prevent oxidant-induced microvascular leukocyte adhesion and attenuate endothelial cell dysfunctions (210), thus limiting inflammation-driven hypertension (211, 212). Overall, it appears that pharmacological modulation of oxidative stress and inflammatory macrophages could represent a viable therapy for the control of hypertension. The HO-1 product CO is also a potent modulator of cellular signaling molecules (i.e. p38 MAPK, ERK1/2, JNK, Akt, NF-κB), which targets mitochondrial activity affecting energy balance and cellular functions (213). Abnormalities of the heme oxygenase/carbon monoxide system have been critically linked to vascular contractility, increased oxidative stress, and imbalanced cellular apoptosis and proliferation in the vascular wall (214). In agreement, Broard et al. have also demonstrated that CO negatively regulates endothelial cell apoptosis, triggering the activation of p38 mitogen-activated protein kinase (MAPK -p38) (215), while pharmacological induction of HO-1 activity decreases blood pressure in spontaneously hypertensive rats (214).

## HO-1 in infections

The role of Hmox-1 and its degradation products is known to have strong implications on the ability to control infections and mitigate their pathological consequences (216). Its relevance in infections is further highlighted by the worrying growth of antibiotic-resistant bacterial strains and the continuous appearance of new viruses. In the present section, we discuss emerging evidence on the crucial role of HO-1 in infectious diseases caused by bacteria and viruses.

### Bacterial infections

*Mycobacterium tuberculosis (Mtb).* HO-1 is expressed in lung tissues and is activated by stress signals, including ROS and inflammatory mediators (217, 218). Its contribution in modulating the host response to Mycobacterium tuberculosis (Mtb) infection and controlling disease progression is still highly controversial. It has been previously reported that Mtb infections can induce HO-1 expression in macrophages *in vitro* and in lung tissue *in vivo* (219). Its activity in macrophages and lungs is associated with increased heme degradation which promotes CO production. The release of this gaseous molecule, acting as regulatory ligand the heme two-component sensor kinases DosS and DosT, induces the complete Mtb dormancy (Dos) regulon (220, 221), thus promoting the bacilli quiescence and the establishment of a latent infection. A published study by Costa et al. supports the role of HO-1 in Mtb survival and replication in the host. The authors observed that SnPPIX-mediated inhibition of HO-1 significantly reduced lung bacterial load, with comparable to the effects achieved with conventional antibiotic therapy. Furthermore, the combination of SnPPIX and antibiotic therapy promoted faster resolution of Mtb infection (222). More recently, the same group found that SnPPIX treatment causes a reduction in intracellular iron availability in activated macrophages, along with an increase in IFNγ-induced NOS2 expression and subsequent NO production, resulting in more effective control of bacterial replication. The authors proposed that HO-1 promotes bacterial survival and proliferation in host cells, through an iron-dependent intracellular mechanism (223). However, contrasting evidence by Chinta et al. indicates that expression of HO-1 in the lungs of patients with Tuberculosis is associated with lower ROS and RNS production. The authors reported in *Hmox1*-deficient mice that lack of HO-1 expression in the damaged lung regions is positively related to an enhanced ROS and RNS production by neutrophils and macrophages, as well as with increased susceptibility to Mtb infections (224).

It should be noted that in the study proposing HO-1 as a promoter of Mtb infections, HO-1 activity was hindered by pharmacological inhibitors, whereas Chinta et al. conducted their experiments on *Hmox1*-deficient mice. These different approaches used may make it more difficult to interpret the mechanisms underlying MtB infection, since HO-1 deficiency was shown to cause important hematopoietic abnormalities (225). However, the protective action of HO-1 has been reported using another Mycobacterium species (i.e. *Mycobacterium avium*), in which mice genetically deficient in HO-1 were found to be more susceptible to infection. In particular, HO-1 deficiency resulted in an accumulation of heme which exerts a cytotoxic effect in infected macrophages, increasing their necrotic death and the consequent release of proinflammatory cytokines (226).

More recently, the importance of the NRF2/HO-1 pathway in determining the outcome of M. avium infection has been demonstrated, thanks to its role in inducing granuloma formation in infected tissue, to counteract bacterial replication (227). Together, these findings might suggest a stage-dependent effect of HO-1, with a cytoprotective role in the initial stages of Mycobacterium infection, which is gradually lost during disease progression, due to massive heme release and subsequent ROS production and oxidative stress (228).

### Salmonella thyphimurium

Infection with S. Typhimurium causes severe gastroenteritis, whose associated inflammatory response is essential for this pathogen to colonize the intestinal tract (229). The intestinal inflammation induced by the infection causes an important dysbiosis, associated with disruption of the colonization barrier, with symptoms that typically include weakness, prostration, fever, and diarrhea, which become particularly problematic in immunocompromised individuals (230). Similarly, to Mycobacterium infection, investigation of HO-1 role in infections with Salmonella highlighted its dual role in the outcome of the disease. A great number of studies unravel the protective role of HO-1 in the infections with this pathogen. Particularly, in an *in vivo* murine model of salmonellosis, HO-1 inhibition with ZnPPIX resulted in an increased apoptosis of liver cells. The mechanistic association of the defective action of HO-1 with a higher susceptibility to *S. thyphimurium* infection, was validated *in vitro*, where HO-1 pharmacological inhibition led to a reduced bacterial killing capacity of macrophages (231). Accordingly, treatment with CoPPIX, an inducer of HO-1, in mice infected with *S. thyphimurum*, may afford protection against the enterocolitis caused by the pathogen (102). In contrast with the above studies, other studies have observed that the ZnPPIX pharmacological inhibition of HO-1 in RAW 264.7 cells, is related to an increased ROS and RNS production that counteract *S.thyphimurum* survival upon infection, suggesting a detrimental HO-1 effect on disease outcome (232).

### Listeria monocytogenes

The Gram-positive bacterium *L. monocytogenes* is borne through contaminated food and infection with this pathogen generally results into mild gastroenteritis. Nonetheless, in elderly or immunocompromised individuals L. monocytogenes infection can lead to severe illnesses, including severe sepsis, meningitis, or encephalitis, that at times may eventually lead to death (233). The literature reports studies both in favor of the protective effect of HO-1 against *L. monocytogenes*, as well as in favor of its infection-promoting action. That said, in response to poly I:C treatment, mice with myeloid-specific deletion of HO-1 infected with L. monocytogenes showed lower levels of IFNγ in the blood, along with improved survival (234). Indeed, myeloid-specific HO-1 is required for the activation of IFN regulatory factor (IRF) 3 and for the production of IFN-β, with the latter increasing the severity of *L. monocytogenes* infection (235, 236). Wang and colleagues observed that L. monocytogenes infection elicits the Tim-3 receptor signaling pathway in macrophages, which inhibits the NRF2 nuclear translocation causing the sequential decrease of HO-1 expression. This mechanism results in an increased susceptibility to *L. monocytogenes in vivo* and *in vitro*, indicating that HO-1 is instead required to limit the bacterial replication during the infection (237). This puzzling scenario of interpretations on the role of HO-1 clearly indicates that we still need to fully understand how to regulate its activity during the different phases of bacterial growth, into the different infectious contexts.

### Viral infections

Recent studies demonstrate that HO-1 has significant antiviral activity against a wide variety of viruses (e.g., influenza virus, respiratory syncytial virus, HIV, hepatitis C virus, hepatitis B virus, enterovirus 71, dengue and Ebola virus) and that this activity may occur through the heme degradation products biliverdin and carbon monoxide, able to respectively inhibit the function of viral proteases and reactive oxygen species production (238). HO-1 indirectly also promotes an antiviral state by inducing activity of IRF3 and the activation of type I IFN antiviral functions (239).

*Human Immunodeficiency Virus (HIV).* Human immunodeficiency virus (HIV). The human immunodeficiency virus (HIV) is the causative agent of acquired immunodeficiency syndrome (AIDS). The available antiretroviral therapies represent the first line of treatment for AIDS, contributing significantly to reducing the number of deaths (240), but to date it appears unlikely that they will exterminate the virus. In this context, several reports dissected the role of HO-1 in HIV infection, associating its activity with the suppression of the virus replication. HO-1 induction by Hemin treatment significantly suppressed infection and viral replication of both monocytes and T cells inoculated with R5, X4, R5X4 tropic viral strains. Such effect was consistent with the inhibition of Tat-dependent activation of long terminal repeat (LTR) viral promoter. Consistently, the HO-1 inhibitor tin-protoporphyrin-IX (SnPPIX) hampered the macrophage resilience allowing the progression of the infection, indicating that the antiviral protective mechanisms in monocytes are mediated by HO-1 activity (241). Along with this, it was reported that bilirubin hinders the protease activity of HIV, thus affecting virus replication (242). Altogether, the above studies suggest the protective role of HO-1 and heme catabolism against HIV infection (242).

### Severe acute respiratory syndrome coronavirus 2

SARS-CoV-2 is the causative agent of COVID-19, that can progress to severe pneumonia with acute respiratory distress syndrome (ARDS) (243). The recent studies of the HO-1 role in the onset of the disease and its progression are still obscure. According to few recent studies, treatments with hemin can attenuate cytokine storms in animal model of sepsis, indicating the putative role of HO-1 in the protection against the cytokine storm syndrome observed in COVID-19 patients (244). Moreover, using a renal cell line (Vero76) infected with SARS-CoV-2 and treated with hemin, it was observed a reduced virus replication, indicating the anti-viral role of HO-1 (245). Singh et al. proposed HO-1 as a protective molecule in the early and late stages of SARS-CoV-2 infections. In fact, the induced expression of HO-1, promotes the expression of type I interferon, which coordinates the anti-inflammatory anti-viral host responses and therefore might protect against the damage from exacerbated oxidative stress (246). However, the role of HO-1 in determining the severity of the infection remains partly to be clarified, as recent the stratification of patients into survivors and non-survivors showed a significant increase of blood HO-1 mRNA levels in the later (247).

HO-1 can be released into plasma by leukocytes, macrophages, smooth muscle cells, and endothelial cells activated by oxidative stress or inflammation (248). Grigorov et al. suggested that serum HO-1 concentrations upon hospital admission of COVID-19 patients could be a useful biomarker for clinical management, proposing that the increase of HO-1 in the early stage of the disease could be beneficial, as it would provide protection against oxidative stress and inflammation (249). However, increased HO-1 expression is not necessarily linked to increased heme catabolism and hemolysis, but could rather be a possible consequence of the inflammatory response as, paralleling the upregulation of blood HO-1, was also reported a significant increment of serum ferritin levels (249), an acute phase reactant that can induce anti-inflammatory response by reducing the damage caused by free radicals (250, 251).

## Conclusions

**Figure 2:**
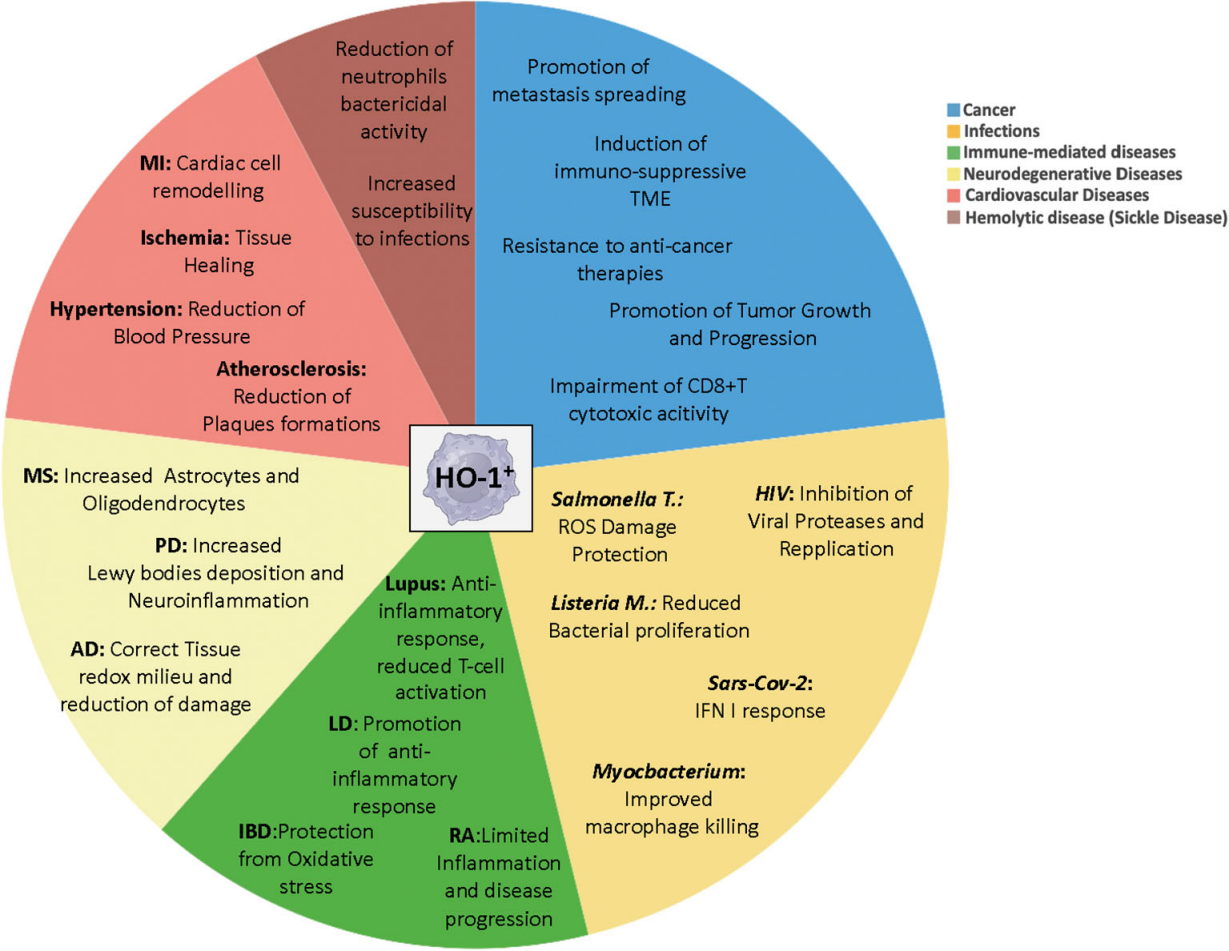
Distinct roles of myeloid HO-1^+^ cells in pathology. Multifaced functions of HO-1^+^ myeloid cells during the onset and progression of several diseases, depending on the pathological contest. MI, myocardial infarction; MS, multiple sclerosis; PD, Parkinson’s Disease; AD, Alzheimer Disease; RA, rheumatoid arthritis; Lysteria M, *Lysteria Monocytogenes*; Salmonella T, *Salmonella Thyphimurium*; IBD, Inflammatory Bowel Diseases; LD, Lung Diseases.

**Figure 3:**
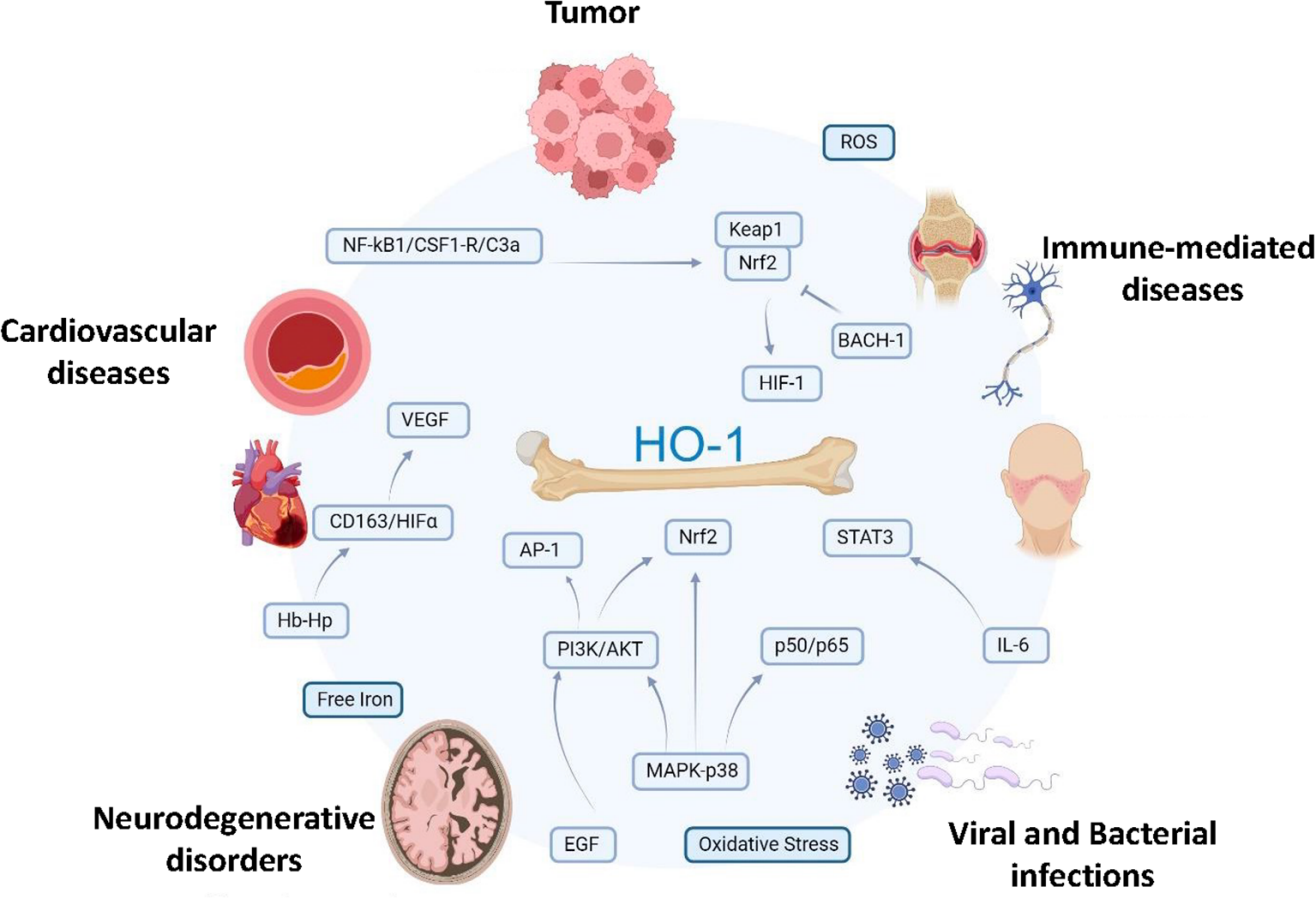
Signaling pathways leading to myeloid HO-1 induction in pathology. The scheme shows potential interrelationships between signaling events promoting transcriptional activation of HO-1 in myeloid cells in different pathophysiological conditions, such as tumors, cardiovascular diseases (ischemic conditions, MI, atherosclerosis, and hypertension) neurodegenerative disorders (AD, PD), immune-mediated diseases (SLE, MS and RA) bacterial and viral infections (*Salmonella thyphimurium, Listeria monocytogenes, HIV* and *SARS-CoV-2*). Several noxious stimuli (i.e. ROS, hypoxia, oxidative stress, hemorrhage/heme, free iron) and inflammatory mediators (i.e. IL-6, VEGF, CSF-1, C3a, EGF, HIF-1a) as well as hemoglobin/haptoglobin through CD163 receptor are known inducers of transcription of HO-1, primarily promoting KEAP-1/Nrf2 nuclear translocation and nuclear export of the transcriptional repressor BACH1. Activation of MAPK/PI3K/AKT, AP-1 and NF-κB by environmental stresses and cytokines has also been implicated in HO-1 activation. HO-1, heme oxygenase-1; MI, myocardial infarction, AD, Alzheimer’s disease; PD, Parkinson’s disease; SLE, systemic lupus erythematosus; RA, rheumatoid arthritis; MS, multiple sclerosis; CSF-1, colony stimulating factor 1; MAPK, mitogen-activated protein kinase; Nrf2, NF-E2 related factor-2; HIF-1a, hypoxia inducible factor; AP-1, activator protein-1; BACH1, HO-1 transcriptional repressor; ROS, reactive oxygen species; PI3K, phosphatidylinositol 3-kinase. Created by biorender.com.

Accumulating evidence point to the multifaced functions of the inducible HO-1 form as critical determinants of disease development since, depending on the pathological contest, its anti-inflammatory and antioxidant properties may elicit beneficial or detrimental effects. While a general protective role of HO-1 has being highlighted in autoimmune, immune-mediated and cardiovascular diseases, development of different infection and cancers benefit from its enzymatic activity (**Figure 2**). Several HO-1 agonists and antagonists have been used to investigate its physiological and pathological roles and to develop new potential therapeutic strategies (**Table 2**). New studies now aim to define the transcriptional mechanisms and differentiation and maturation pathways that regulate HO-1^+^ myeloid populations in pathology. Transcriptional (i.e. Nrf2, Maf, AP-1, p50 NF-kB) (7, 252) and epigenetic events (83), as well as microenvironmental conditions (i.e. hypoxia) (253) finely tune *Hmox-1* gene expression. Above this, new emergency pathways of hematopoiesis display the capacity to promote expansion of circulating monocytes and macrophages endowed with HO-1 activity (7) (**Figure 3**). Understanding the interconnection between local (microenviroment) and remote (emergency myelopoiesis) mechanisms of HO-1 regulation could improve our ability to therapeutically target its functions in disease, also considering its dynamic and stage-dependent activity observed in distinct pathological contexts. Furthermore, making the biology of HO-1 even more intricate, a body of evidence indicates that the anti-inflammatory role of HO-1 predominates in the early stages of the experimental inflammatory diseases. Indeed, hemin-like compounds or adoptive transfer of hemin-activated macrophages reduced caerulein-induced pancreatitis, serum amylase and lipase, decreased pancreatic trypsin generation, and protected from lung injury (254), while activation of the NRF2/HO-1 pathway, through administration of Cobalt protoporphyrin IX (CoPP), induced macrophage differentiation toward a Marco^hi^Hmox1^hi^ anti-inflammatory erythrophagocytic phenotype, contributing to an overall decreased inflammatory profile in a murine model of colitis (255). However, the anti-inflammatory action of HO-1-inducing compounds observed in these models was evidenced mainly during the onset phase of the inflammatory response, while it was found to be lacking when induced after disease onset.

**Table 2:** HO-1’s agonists and antagonists.

Disease	HO-1 agonist or antagonist		Mechanism	Impact on disease/therapy
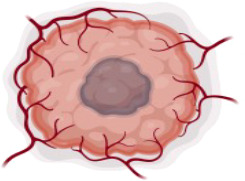 **Cancer**	*LL2 and PDAC*	Antagonist (SnMP) (49, 53)	Suppression of immunosuppressive role of TAMs	Decreased tumor growth and progression
	*4T1*	Antagonist (SnMPIX) (43, 63)	Abrogation of TAMs pro-angiogenic activity	Reduced metastatic spread
	*MN/MCA1;B16/F10*	Antagonist (ZnPPIX) (7)	Reduced immunosuppressive myelopoiesis	Increased activity of anti-PD-1 immunotherapy
	*MMTV-PyMT*	Antagonist (SnMPIX) (22)	Improved cytotoxicity of CD8+ T cells	Increased antitumor activity of 5-fluorouracil
	*GBM*	Antagonist (ZnPPIX;OB24) (46, 73)	Reduced levels of PD-L1/PD-1 and IL-10	Recovered of pro-inflammatory, antitumor TME
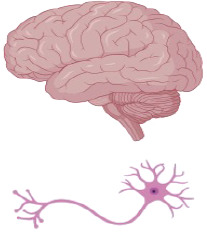 **Neurodegenerative disorders**	*Multiple Sclerosis*	Agonist (122, 125)	ROS damage	Increased levels of astrocytes and oligodendrocytes
	*Alzheimer’s disease*	Agonist (111, 127)	Promotion of correct tissue redox milieu	Reduced severe state of both inflammation and tissue damage
	*Parkinson’s disease*	Antagonist (114, 117, 133)	Reduced iron content in Lewy bodies	Mitigation of neuroinflammation, cerebral injury and α-synuclein protein sedimentation
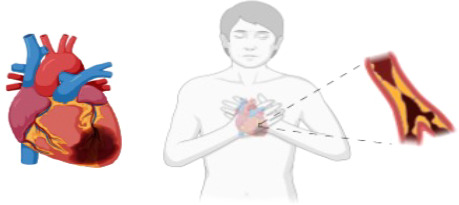 **Cardiovascular** **diseases**	*Ischemia*	Agonist (112, 160, 165)	ROS damage	Tissue Healing
	*Myocardial infarction*	Agonist (174, 175)	Angiogenesi	Cardiac remodeling and preserving the ventricular rupture
	*Atherosclerosis*	Agonist (181–184, 193)	Reduced Foam cells accumulation	Absence of atherosclerotic plaques formation
	*Hypertension*	Agonist (207–213)	Reduction of inflammation-driven hypertension	Decreases blood pressure
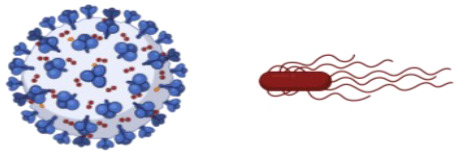 **Viral and Bacterial** **infections**	*Mycobacterium*	Antagonist (SnPPIX) (220–222)	Reduced intracellular iron availability in macrophages	Inhibition of bacterial survival and proliferation
	*Salmonella thyphimurium*	Agonist (224)	Reduced necriotic death of macrophages	Cytoprotective role in the initial stages of infection
	*Listeria monocytogenes*	Agonist (CoPPIX) (101)	Improved bacterial killing by macrophages	Decreased susceptibility to bacterial infection
	*HIV*	Antagonist (ZnPPIX) (230)	Increased ROS and RNS production	Reduced bacterial survival
	*SARS-CoV-2*	Agonist (235)	Promotion of IFN I	Reduced bacterial proliferation
		Agonist (235) (Hemin; CoPPIX)	Inhibition of viral proteases and reactive oxygen species production	Inhibition of Viral replication
		Agonist (244) (Hemin; CoPPIX)	Promotion of IFN I	Repression of exacerbated oxidative stress and promotion of anti-inflammatory response
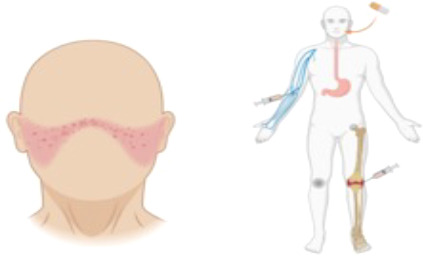 **Immune-mediated diseases**	*Lupus erythematosus*	Agonist (252)	Polarization of macrophages towards an M2-like profile	Reduced T cell activation and increased Treg differentiation
	*Rheumatoid arthritis*	Agonist (91)	Limited inflammation and toxicity	Decreased disease progression
	*IBD*	Agonist (99)	Reduction of Th17 cell number; increased Tregs	Reduced inflammation
	*Lung diseases*	Agonist (107–110)	Activation of IFN I response	Resolution of airways inflammation

Their effects are reported in the different pathological contexts, including Cancer, Neurodegenerative disorders, Cardiovascular diseases, Viral and bacterial infections, and Immune-mediated disorders. The table includes studies and experimental models cited in the manuscript. CoPP, cobalt protoporphyrin IX; ROS, Reactive oxygen species; IFN, interferon; BMDM, bone-marrow derived macrophages; SnMP, tin mesoporphyrin; ZnPPIX, zinc protoporphyrin IX; 4T1, model of breast adenocarcinoma; LL2, Lewis lung carcinoma; PDAC, pancreatic ductal adenocarcinoma; MN/MCA1, fibrosarcoma; B16/F10, melanoma; MMTV-PyMT spontaneous mouse model of breast cancer; GBM, glioblastoma.

As discussed above, HO-1 deficiency causes important hematopoietic abnormalities (225) and has an impact on the regulation of innate and adaptive immune responses. Consistently, genetic alteration of HO-1 gene is associated with important hematopoietic and organ dysfunctions. Sickle cell disease (SCD) is an autosomal recessive pathology generated by a genetic point mutation at the level of β-globin locus. The effects generated by this mutation results in an abnormal form of hemoglobin, characterized by red blood cell membrane rigidity and consequently hemolysis (256). Among the pathological complications, SCD bearing individuals shown more susceptibility to precise bacterial infections, including Salmonella, Streptococcus Pneumoniae and Hemophilus Influenzae, resulting in a potent risk factor for invasive bacterial infections, where a splenic dysfunction has been considered the main leading cause (257). Further, tissue iron overload and anemia were previously reported in a human patient and mice lacking HO-1 (225). It was found that resident splenic and liver macrophages were mostly absent in HO-1-deficient conditions and that HO-1^−/−^ macrophages died of exposure to heme released on erythrophagocytosis. The release of heme, following the rupture of HO-1^−/−^ macrophages, caused further tissue inflammation, with initial splenic enlargement progressing to red pulp fibrosis, atrophy, and functional hyposplenism, that recapitulated the asplenia of an HO-1–deficient patient. This genetic evidence further highlights the key role of HO-1-expressing erythrophagocytes in tissue homeostasis and iron redistribution.

It appears clear that the therapeutic potential of HO-1 in mediating the resolution of inflammation and tissue homeostasis may reside in specific phases of the inflammatory cascade and the regulation of hematopoietic output, thus calling for further study to fully characterize its therapeutic potential in different pathological contexts.
